# Identifying the Information Needs and Format Preferences for Web-Based Content Among Adults With or Parents of Children With Attention-Deficit/Hyperactivity Disorder: Three-Stage Qualitative Analysis

**DOI:** 10.2196/47409

**Published:** 2023-09-11

**Authors:** Danielle A Scholze, Melissa M Gosdin, Susan L Perez, Julie B Schweitzer

**Affiliations:** 1 MIND Institute University of California, Davis Sacramento, CA United States; 2 Department of Pediatrics University of California, Davis Sacramento, CA United States; 3 Center for Healthcare Policy and Research University of California, Davis Sacramento, CA United States; 4 Department of Kinesiology and Health Science California State University Sacramento, CA United States; 5 Department of Psychiatry and Behavioral Sciences University of California, Davis Sacramento, CA United States

**Keywords:** ADHD, pediatric, adult, mobile phone, developmental disorder, neurodevelopmental, mental disorder, information-seeking, information need, attention deficit disorder, hyperactive, hyperactivity, interview, focus group, think-aloud, web-based, online content, user experience, information behavior, web design

## Abstract

**Background:**

Attention-deficit/hyperactivity disorder (ADHD) is a highly prevalent childhood and adult behavioral disorder. Internet searches for ADHD information are rising, particularly for diagnosis and treatment. Despite effective ADHD treatments, research suggests that there are delays in seeking help for ADHD. Identifying ways to shorten delays is important for minimizing morbidity associated with ADHD. One way to shorten these delays is to improve internet health information resources. Research shows that parents of children with ADHD feel that much of the information available is technical and not tailored for their child’s needs and verbal instructions given by health care providers were too pharmacologically focused with limited information about how to manage and support ADHD symptoms in daily living. A majority of parents search the internet for general and pharmacological information for ADHD and prefer web-based resources for learning about ADHD, but web-based resources may be inaccurate and of low quality. Ensuring accurate information through the internet is an important step in assisting parents and adults in making informed decisions about the diagnosis and treatment of ADHD.

**Objective:**

Although a great deal of information regarding ADHD is available on the internet, some information is not based on scientific evidence or is difficult for stakeholders to understand. Determining gaps in access to accurate ADHD information and stakeholder interest in the type of information desired is important in improving patient engagement with the health care system, but minimal research addresses these needs. This study aims to determine the information needs and formatting needs of web-based content for adults with ADHD and parents of children with ADHD in order to improve user experience and engagement.

**Methods:**

This was a 3-phase study consisting of in-depth phone interviews about experiences with ADHD and barriers searching for ADHD-related information, focus groups where participants were instructed to consider the pathways by which they made decisions using web-based resources, and observing participants interacting with a newly developed website tailored for adults with potential ADHD and caregivers of children who had or might have ADHD. Phase 1 individual interviews and phase 2 focus groups identified the needs of the ADHD stakeholders related to website content and format. Interview and focus group findings were used to develop a website. Phase 3 used think-aloud interviews to evaluate website usability to inform the tailoring of the website based on user feedback.

**Results:**

Interviews and focus group findings revealed preferences for ADHD website information and content, website layout, and information sources. Themes included a preference for destigmatizing information about ADHD, information specific to patient demographics, and evidence-based information tailored to lay audiences.

**Conclusions:**

ADHD stakeholders are specifically seeking positive information about ADHD presented in a user-friendly format.

## Introduction

### Background

Attention-deficit/hyperactivity disorder (ADHD) is a chronic neurodevelopmental disorder characterized by developmentally distinct levels of impulsivity, hyperactivity, and inattention. ADHD is the most prevalent behavioral disorder affecting children, with estimates ranging from 5% to 11%, and is one of the most common psychiatric disorders affecting adults (4.4%) [1-3]. Based on a survey of parents, ADHD diagnosed by a health care provider has increased by 42% from 2003 to 2011 [2]. Although the onset of ADHD symptoms occurs during childhood, approximately two-thirds of children with ADHD are symptomatic into young adulthood [4]. ADHD is associated with many negative outcomes including increased rates of grade retention and high school dropout rates [5]. Functional impairments continue into adulthood as well. A Norwegian study found that only 22.2% of those with ADHD had regular work as their source of income compared with 72% in the general population. In addition, it found a negative correlation between the age of first stimulant use and occupational outcomes [6]. Rates of mental health comorbidities such as anxiety and depression are also higher in both children and adults with ADHD when compared to counterparts without ADHD. Based on a review study completed in 2012, the economic burden of ADHD in the United States is estimated to be between US $143 and US $266 billion, with the majority being incurred during adulthood due to ADHD-associated productivity and income losses [7]. In addition, a systematic review completed in 2012 showed that the long-term outcomes of those treated for ADHD were improved over those without treatment [8]. When considering all these facts, it is apparent that timely diagnosis and treatment of ADHD are essential to maximize long-term functioning and minimize costs to society.

Although significant benefits are seen with the treatment of ADHD, research suggests that the delay in seeking help for ADHD is on average 3.96 years in children and 13 years in adults [9,10]. Finding ways of shortening these delays is important for minimizing morbidity associated with ADHD. Interestingly, concerns exist that ADHD may simultaneously be overdiagnosed and overtreated based on extremely high rates in certain communities [11]. The controversies surrounding ADHD not only affect the patients and their families but also those undiagnosed or misdiagnosed as well. Social stigma, lack of knowledge, and misinformation about ADHD are barriers to obtaining evidence-based treatment [12]. ADHD also appears to be uniquely trivialized in the media and pop culture, with jokes and even songs about ADHD being a common occurrence. The combination of public stigma, skepticism, and misinformation in the community contributes to delayed diagnosis and treatment. These controversies directly impact the mental health of patients, their families, and their communities.

Parents are the primary group of caregivers seeking information about ADHD diagnosis and treatment. Research shows that parents of children with ADHD feel that much of the information available is technical and not tailored to their child’s needs. In addition, they felt as if the verbal instructions given by health care providers were too medicine-focused with limited information about how to manage and support ADHD symptoms in daily living [13]. Over 80% of parents search the internet for general and medication information for ADHD and 70% of parents prefer to use web-based learning programs about ADHD [14]. While research shows that parents of children with ADHD have a significant increase in ADHD knowledge after accessing high-quality and credible websites [15,16], only one-third of parents trust the information obtained on the internet [17]. It has been found that the majority of websites providing information about mental disorders are of poor quality [18], with only 12%-54% of ADHD websites in agreement with recommended evidence-based treatment guidelines [19]. A study of ADHD websites in Spanish found that the majority of websites contained treatment information categorized as low quality [15]. Interpreting ADHD information is further complicated by the distribution of untested “treatments” marketed on the internet and television. In addition, the impact of ADHD on adults is now widely recognized, but there is essentially no research looking at the information-seeking behaviors and information needs of adults with ADHD. This is a large gap in the ADHD literature that needs to be addressed.

Although the impact of ADHD on children, adults, and society is well described in the literature, there continues to be a significant delay in seeking help for ADHD. Stigma, misinformation, and lack of information contribute to this delay. With the internet becoming a rapidly growing source of health care knowledge, it is essential to determine ways of utilizing its power to educate large populations. Communicating accurate information through the internet is an important step in assisting patients and adults in making informed decisions about the diagnosis and treatment of ADHD. This study aims to address gaps in knowledge access through interviews and focus groups with the stakeholders seeking this information, including adults with ADHD.

### Objective

The objective of this study was to improve the quality and usability of a website based on the information and user needs of adults and parents of children with ADHD in order to ensure individuals in need of ADHD information have accurate, easy-to-access information in the hopes of improving knowledge acquisition, user experience, and self-efficacy in relation to ADHD. We achieved this goal by identifying the information needs and preferences of stakeholders (family members and children or adults with ADHD) around ADHD, gathering stakeholder feedback to develop a website meeting these needs and preferences, and assessing the ways in which users interact with and access information on the website.

## Methods

### Setting and Participants

Participants were recruited through a search of existing networks of ADHD parents (eg, a local ADHD support group, Children and Adults with Attention-Deficit/Hyperactivity Disorder [CHADD]), advocates, educators, and primary care providers, including public steering committees and the Institute’s recruitment volunteer registry.

### Data Collection

A qualitative framework was used to explore the informational needs of adults with ADHD and parents of children with ADHD for the purpose of designing a website dedicated to providing educational resources to the general public. This was a 3-phased study. The study team consisted of experts in qualitative inquiry, health services researchers, a sociologist, a behavioral pediatrician, and a licensed psychologist with expertise in ADHD. The research team interviewed individual participants to gather data regarding ADHD information needs, moderated focus groups to gather data regarding decision-making strategies and website format preferences, and guided participants in a think-aloud interview utilizing the newly developed website.

Phase 1 consisted of in-depth phone interviews where participants were asked to discuss their experiences with ADHD, barriers they encountered searching for ADHD-related information (eg, definition, symptoms, and treatments), and information preferences (eg, information source, content, and layout).

Phase 2 consisted of focus groups where participants were instructed to consider the pathways by which they made decisions (in the presence or absence of the needed information) as well as the important aspects of information, points of access, and decision-making points. During this phase, participants were also guided through an activity to sketch preferred layouts for websites that they visited when seeking information related to ADHD. Participants were asked to share their sketches to highlight the attributes of the layouts that made navigating the website and accessing information easier. Using the findings from phase 1 and phase 2, a website was developed by content experts at a university, neurodevelopmental center.

In phase 3, we observed participants interact with the newly developed website to understand the ways in which our target audience used the website. Interviews consisted of participants navigating through the newly developed website while thinking aloud. Participants’ internet searches were recorded using screen capture software (Camtasia, version 9.0; TechSmith).

### Data Analysis

**Figure 1:**
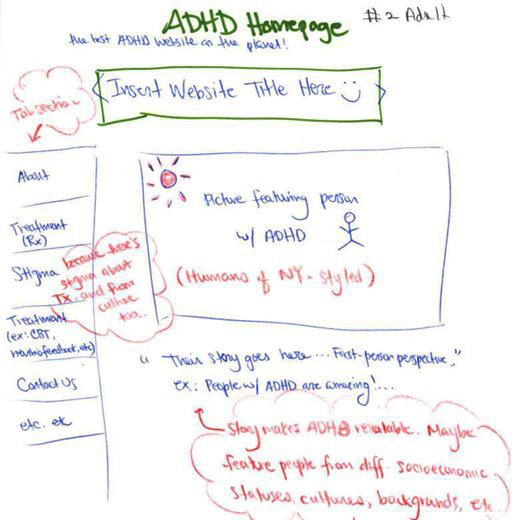
Example of preferred website layout drawn by focus group participant. ADHD: attention-deficit/hyperactivity disorder.

**Figure 2:**
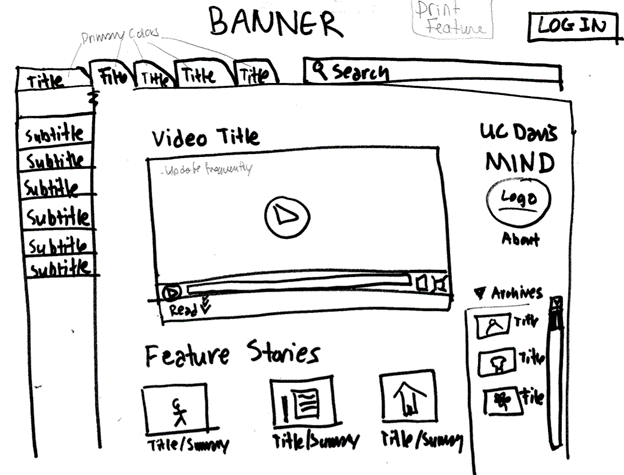
Wireframe compiled from multiple focus group participants’ input.

**Figure 3:**
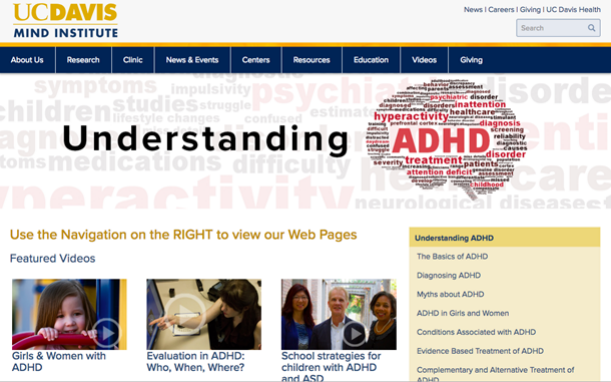
Screenshot of user-informed website developed following phase 1 and phase 2 of the study. ADHD: attention-deficit/hyperactivity disorder.

The investigators transcribed the interviews and used thematic analysis to identify common, data-driven themes and to describe patterns found in the data. Phase 2 participant sketches of preferred website layouts (Figure 1) were compared for consistent patterns and preferences for website attributes, layouts, and features. Individual participant sketches were compiled to form a wireframe diagram (Figure 2). Findings from phase 2 were then used to develop both the content and design of the website (Figure 3). Phase 3 screen capture recordings were analyzed for website navigation patterns (eg, identifying where participants are first drawn when entering the website), areas where participants became confused or experienced challenges finding relevant information, and website features participants particularly liked or disliked.

### Ethics Approval

This study was reviewed and approved by the University of California, Davis Internal Review Board (project #:816998). All participants signed an informed consent outlining what their participation involved, the purpose of the study, and their rights and role as a participant. During the session, the study team reviewed the informed consent document to ensure participants understood the informed consent process. Recordings from the interviews and think-out-loud exercises were deleted, transcribed, and deidentified. Recordings were stored in a secure file folder that only the study team had access to. Parents of children with ADHD and adults with ADHD participating in the focus groups received a US $25 Amazon gift card, educators received a US $50 Amazon gift card, and clinicians received a US $100 Amazon gift card.

## Results

In total, 15 in-depth interviews were conducted in phase 1 illuminating participants’ needs for stakeholder-specific and evidence-based information.

### Stakeholder Specific Information

All persons interviewed indicated that there is a dearth of information regarding ADHD and that a centralized source that could provide quality information (eg, trustworthy, up-to-date, and reliable) is needed. All stakeholders expressed that information should be tailored to individual interests and needs. For example, a website includes tabs linked to information for specific populations.

Regardless of stakeholder type, interview participants stated that information should be presented and framed by different stakeholders’ perspectives. For example, information regarding the signs and symptoms of ADHD should be presented by age and stage of development. A mother of 2 children with ADHD stated that parents often “want to know what are the symptoms, how to get diagnosed, and a checklist.” Stakeholders also expressed a need for strategies to guide discussions about ADHD with nonclinicians. Specifically, several parents expressed the need for education on communicating with teachers, family, and friends about their child’s ADHD. Clinicians requested information about how to talk to parents about ADHD, including an ADHD handout explaining basic information about ADHD.

### Evidence-Based Information

Evidence-based research includes both contemporary journal papers and summaries of studies that are straightforward and easily understood by lay audiences. Participants expressed a specific need for information about medication and its management, such as dosing in various contexts (eg, weekends vs school days), and side effects were also requested. Parents expressed a need for links to assessment tools and a list of resources including websites, therapists, community services, and readings. Almost all participants stated that if the website were linked to a locally known institute for neurodevelopmental disorders at a university or other “trustworthy” sources that it would be seen as reputable.

Two focus groups conducted (N=13 participants) in phase 2 identified participants’ information preferences and seeking strategies. Participants are seeking out information that is destigmatized and tools to identify ADHD-related behaviors and relevant support resources.

### Destigmatized Content

Adults with ADHD frequently expressed wanting to read or watch videos of individuals who have overcome the challenges associated with ADHD, while highlighting the many unique talents and skills of those with ADHD. A female partner of an adult male who was diagnosed with ADHD said, “there are a lot of great things that come from having ADHD and I want him to see that.”

Adult participants were more likely than the parental group to express concern regarding the stigma associated with ADHD and expressed negative characteristics are typically associated with ADHD. An adult participant explained the stigma associated with ADHD, “having ADHD is like running in mud, you are putting forth a lot of effort but making little progress and it’s important to know that you are not broken but that you just learn differently.”

### Tools to Identify Behaviors and Support Resources

Parents with children who have or are suspected of having ADHD expressed a desire for 3-minute videos on how to recognize and address specific behavior associated with ADHD and ways of addressing such behaviors, information about co-occurring disorders, and short videos focusing on a variety of learning techniques.

Both groups of participants reported wanting information about ADHD support groups and community resources that are age-appropriate and concise information that is easy to understand and presented in a clear, colorful format. Additionally, both groups expressed the need for having access to evidence-based practices, techniques, and tools provided by a trusted source.

The research team conducted 6 think-aloud interviews to assess the newly created website’s user-friendliness and usefulness with individuals who self-reported a diagnosis of ADHD or identified as being a parent of a child with ADHD. The research team identified factors that improve the website’s intuitive design, the ways in which participants attribute of trustworthiness of the website and information, and website layouts that improve website impressions.

### Intuitive Design

When prompted to search for information about ADHD, most participants searched the top portion of the website (menus representing the Institute and its university) that were not part of the website designed for this study. Many expressed frustrations that they could not find what they were looking for because they were intuitively drawn to the main tabs at the top of the page. For example, when searching for scientific information on ADHD, some went to the research tab and found current clinical trials rather than peer-reviewed ADHD resources.

A few participants went straight to the search box to look for information or resources on ADHD while others said they overlooked the search box as it did not stand out on the page. Often those who found and used the search box eventually found the information that they were looking for.

When scrolling down the page looking for information on how to cope with ADHD, for example, some said if they were home alone, they would have left the page and used a search engine to locate the information they were seeking. This occurred for two primary reasons (1) participants were searching for the exact words “coping with ADHD” and could not immediately find this information or (2) they did not take the time required to read an entire document to find the information. Some participants reported they were searching for more bulleted lists. However, there are bulleted lists on the website, so these frustrations may be more of a design issue rather than a content problem.

Participants also commented on specific design and layout elements that they perceive as helpful to website navigation such as font type, size, color, and background. One adult participant reported that “soft and inviting” fonts such as Comic Sans are desirable in addition to darker color fonts against light backgrounds.

### Trustworthiness of Information Sources

Many participants expressed confidence in the information provided on the website because of its association with the local university and more specifically an institute well-known for its work in neurodevelopmental disorders in the region. There were a couple of participants who were not as concerned with the quality of the information provided on the website, stating that “if you google symptoms of ADHD you will find the same information.” This implies that there is little or no variation in content, especially concerning “basic information.”

### Overall Impressions

In general, participants were drawn to easy-to-understand bullet lists, colorful graphics, and videos. Participants experienced challenges with using the search function and recommendations for improvement included the need for a better search feature and clear drop-down menus.

Throughout all 3 phases of the study, there were overarching themes of participants’ desire for ADHD content that is current evidence-based information, used a strength-based approach to addressing ADHD, and included other resources and tools as well as website design that includes interactive infographics and videos, and is user-friendly for navigation (see Textbox 1). In addition to these general themes, participants sought out specific information about the developmental course of ADHD, strategies for talking with others about ADHD, techniques to foster learning, and management of behavioral difficulties.

**Emerging themes throughout the study.**

**Content specific**

Current evidence-based information:

- Peer-reviewed papers
- Links to additional resources or websites

Strength-based approach:

- Address attention-deficit/hyperactivity disorder (ADHD)–related stigma
- Career options for those with ADHD
- Positive attributes (humor, creativity)

Resources:

- Local physicians, counselors, and support groups
- Links to ADHD blogs, books, webinars, and podcasts
- List of helpful smartphone apps
- Diagnostic assessments

**Design specific**

Interactive infographics:

- Chat feature
- Bulletin boards

Videos:

- Success stories
- Neurodiversity

User-friendly navigation:

- Tabs or menus
- Ample white space
- Bullet points
- Tool kit format

## Discussion

### Key Findings

The objective of this study was to determine the information and formatting preferences of ADHD stakeholders to build a user-informed website. Participants indicated both strengths and areas of improvement regarding ADHD websites.

This study addressed the question of how individuals might assess whether the information is trustworthy and credible. Participants in this study knew to look for information from credible sources, such as peer-reviewed literature or connections with credible institutions, and requested evidence-based information from peer-reviewed journals that were easily understood by a lay audience. Individuals seeking information about ADHD have a preference for a higher level of credibility of information and know how to identify highly credible information.

The presentation format of ADHD-related content is as important as the information itself must be user-friendly. Participants emphasized the potential impact website design has on whether the information presented is accurately understood. The majority of participants reported that regardless of the quality of available content, a website must contain certain features such as drop-down menus that are located at the top of the page, a search menu, interactive design, and information presented through infographics and videos. A commonly expressed concern about many existing ADHD websites is that stakeholder preferences are not always factored into the design. For example, a balance between what one could consider attention-grabbing and distracting must be considered when presenting information in the form of images. The colors, font type, and size mattered to the majority of participants.

Although many of the information needs and format preferences expressed by ADHD stakeholders are similar to those associated with other health conditions, there were themes that appeared to be unique in this population. One of the most striking findings was that the majority of adult participants with ADHD expressed that they were seeking destigmatized and positive information about ADHD, specifically success stories and information on strengths associated with ADHD. This finding reinforces the importance of focusing not only on appropriate evaluation and evidence-based treatment of ADHD but also on how differences can be used in a beneficial way. Interestingly, social stigma was not a theme specifically raised by the parents of children with ADHD in this study. That being said, a study from 2010 showed that 44% of parents were concerned about societal views of the ADHD label and 40% felt isolated and rejected [20]. In addition, a study in 2003 showed that 39% of parents reported stigma as a barrier to getting help for their child with ADHD [21]. Mental illness stigma, such as that associated with ADHD, has well-documented effects on not only patients but also system resources and provider behavior [22]. Providing destigmatized information on the internet is an important step to decreasing stigma in society.

A second theme focused on obtaining evidence-based and up-to-date treatment information in a format that is easy to understand. Participants specifically requested peer-reviewed literature and evidence-based treatments but struggled to find that information in an easily understood format. Unfortunately, people are overwhelmed with new “treatments” for ADHD, but it is considerably challenging to separate those supported by appropriate evidence versus those that are not. This has been shown to be one of the barriers to evidence-based treatment of ADHD [12].

### Limitations

This study was limited geographically, and the results may not be generalizable to other regions because of the availability of resources specific to this region. The sample size for each phase was small. All participants had an educational level beyond high school and thus, may not represent individuals with less education. Participants involved were also sophisticated enough to seek out support groups for ADHD (ie, CHADD) and a university center for neurodevelopmental disorders; therefore, individuals with less knowledge of how to access these resources may not be well-represented in this sample. Furthermore, this study is a snapshot of the information-seeking experiences of individuals during a specific time period and since then there may have been other resources made available to these individuals. Last, this study was limited to English-language health information resources only.

### Conclusions

This study explores the process of creating a user-informed ADHD website. ADHD stakeholders (adults with ADHD and parents of children with ADHD) provided suggestions pertaining to both desired content and the design of a website dedicated to providing a wide spectrum of ADHD content in a user-friendly format. Participants identified ways to best address the unmet informational needs of ADHD stakeholders including providing evidence-based content that can easily be understood by nonacademic audiences, success stories from those who have ADHD, awareness of neurodiversity, nonstigmatized perspectives, and access to medical and educational resources. Participants also provided feedback regarding how to best design a website devoted to ADHD information sharing including limited text, bullet points, an interactive format, a search feature, and clearly identifiable menu options. Creating a dedicated ADHD website informed and tested by a variety of stakeholders can advance public knowledge while increasing the self-efficacy needed to improve the overall quality of life of those impacted by ADHD.

Evaluation of the accuracy of information on the internet provides important insight into the type of information encountered but does not provide insight into how health information is used by the lay public. Previous studies assessing the influence of internet health information have not inquired as to how information influenced decisions and stopped short of assessing the quality of the information used [23,24]. Given the specific needs and preferences of individuals seeking information about ADHD, future research is needed to specifically understand the influence of ADHD websites on knowledge acquisition, self-efficacy, and patient activation. To our knowledge, this is the first study to address the information needs of adults with ADHD, thus additional research specifically into the needs of adults with ADHD is also essential.

This study introduced the concept that many ADHD information seekers have a high level of sophistication, specifically looking for evidence-based practices and peer-reviewed literature about ADHD. Future educational websites and materials should focus on providing higher-level information in formats that are accessible to all information seekers. This study highlighted the importance of providing destigmatized information about ADHD. Utilizing asset-based messaging can help provide positive information about ADHD and techniques to use the unique traits of those with ADHD. Although future research in this area is critical to further understand the information needs of those affected by ADHD, many improvements currently can be made that will allow ADHD stakeholders to have better access to the information and decrease the stigma that continues to be associated with ADHD.

## Abbreviations

ADHD: attention-deficit/hyperactivity disorder
CHADD: Children and Adults with Attention-Deficit/Hyperactivity Disorder

## Data Availability

The data generated and analyzed during this study are not publicly available due to potentially identifying information revealed during the interview or focus group but are available from the corresponding author upon reasonable request.
